# *C. elegans*-based chemosensation strategy for the early detection of cancer metabolites in urine samples

**DOI:** 10.1038/s41598-021-96613-z

**Published:** 2021-08-24

**Authors:** Enrico Lanza, Martina Di Rocco, Silvia Schwartz, Davide Caprini, Edoardo Milanetti, Giuseppe Ferrarese, Maria Teresa Lonardo, Luca Pannone, Giancarlo Ruocco, Simone Martinelli, Viola Folli

**Affiliations:** 1 Istituto Italiano di Tecnologia, Center for Life Nano Science, Rome, 00161 Italy; 2 Department of Oncology and Molecular Medicine, Istituto Superiore di Sanità, Rome, 00161 Italy; 3 Department of Biochemical Science “A. Rossi Fanelli”, Sapienza Università di Roma, 00185 Rome, Italy; 4 Department of Physics, Sapienza Università di Roma, Rome, 00185 Italy; 5 Department of Surgery, Ospedale M. G. Vannini, Rome, 00177 Italy; 6 Genetics and Rare Diseases Research Division, Ospedale Pediatrico Bambino Gesù, IRCCS, Rome, RM 00165 Italy

**Keywords:** Olfactory receptors, Breast cancer

## Abstract

Chemosensory receptors play a crucial role in distinguishing the wide range of volatile/soluble molecules by binding them with high accuracy. Chemosensation is the main sensory modality in organisms lacking long-range sensory mechanisms like vision/hearing. Despite its low number of sensory neurons, the nematode *Caenorhabditis elegans* possesses several chemosensory receptors, allowing it to detect about as many odorants as mammals. Here, we show that *C. elegans* displays attraction towards urine samples of women with breast cancer, avoiding control ones. Behavioral assays on animals lacking AWC sensory neurons demonstrate the relevance of these neurons in sensing cancer odorants: calcium imaging on AWC increases the accuracy of the discrimination (97.22%). Also, chemotaxis assays on animals lacking GPCRs expressed in AWC allow to identify receptors involved in binding cancer metabolites, suggesting that an alteration of a few metabolites is sufficient for the cancer discriminating behavior of *C. elegans*, which may help identify a fundamental fingerprint of breast cancer.

## Introduction

The discovery of cheap and non-invasive diagnostic strategies for the early detection of cancer is an urgent priority. Cancer is a disease that deeply alters the metabolome of the organism, potentially introducing its own characteristic waste products in biofluids^1,2^. It has been reported that cancer tissues exude volatile compounds, and taking advantage of the extraordinary sense of smell of dogs and mice, previous studies allowed to demonstrate the existence of yet unknown cancer-specific odorants in biological fluids^3,4^. Animals perceive as odors a large variety of volatile molecules through olfactory perception. The ability to respond adaptively to any chemical alteration of the surrounding world is of pivotal importance for life and health in most animal species^5^. Indeed, odors serve animals as environmental trackers to localize food and water sources, for nesting, and to discriminate conspecifics (pheromones^6^) from individuals of other species (allelochemicals^7^). Different compounds or different ratios of the same compound result in a specific odor fingerprint that may modulate such behaviors.

Odor sensing is mediated by a large family of proteins, known as G-protein coupled receptors (GPCRs)^8^. These are seven-transmembrane domain proteins that upon binding their ligands, activate G-proteins, which in turn initiate intracellular signaling cascades. GPCRs are mainly expressed in the cilia of the olfactory neurons (ORNs)^9^. As yet, it is widely accepted, although with some exception^10^, that each olfactory neuron in mammals expresses uniquely one kind of olfactory receptor^11–13^. The segregation of GPCRs on distinct olfactory cells ensures odor discrimination when the signals from functionally identical neurons are integrated into the olfactory bulb. On the contrary in *C. elegans*, each olfactory neuron presents multiple types of GPCRs on its membrane. *C. elegans* is a nematode widely studied as a model organism in different fields thanks to many properties, which include ease of cultivation, invariant cell number, genetic tractability, and optical accessibility of its cells. In the past years, these advantages have been exploited, leading, for example, to the reconstruction of its entire cell lineage^14,15^, and of its nervous system^16–19^, although the latter is still lacking some key information^20^. Interestingly, despite the large number of genes encoding olfactory receptors (more than 1,000 including gustatory receptors)^21,22^, this nematode possesses a relatively small number of chemosensory neurons, namely, 32, three of which are specialized to sense volatile molecules. Each olfactory neuron in *C. elegans* is therefore likely to detect a wider range of odorants if compared to mammalian ones^23,24^. As a consequence, the combinatorial complexity of the odorant-receptor pair is extremely high in the nematode. Furthermore, the same GPCR can be expressed on distinct cells^22^ and the same odorant can bind different GPCRs on functionally different neurons and, depending on its concentration, it may elicit opposite behavioral outcomes^25^. To date, the majority of the behavioral olfactory assays reported in *C. elegans* are designed for single substances, and responses have been assessed for a large variety of either volatile or soluble molecules including alcohols, ketones, aldehydes, esters, amines, sulfhydryls, organic acids, aromatic and heterocyclic compounds^24,26^. Many of the substances mediating attraction are natural metabolic products of bacteria^27,28^, the food source of the nematode. However, the environmental stimuli *C. elegans* is exposed to are mostly complex mixtures of chemical compounds associated with competitive responses. Different ratios of attractive and repulsive components of a mixture can asymmetrically activate the neuronal architecture involved in the sensation of odors. As a result, *C. elegans* preference between two odors can be inverted in the presence of a third one^29,30^. This highlights the importance of the chemical background on the *C. elegans* olfactory decision-making mechanism^31^.

Recently, it was demonstrated that *C. elegans* displays attractive chemotaxis towards cancer urine samples while it is repelled by control samples^32^ in a highly accurate way. The ablation of olfactory neurons and the study on G protein $\alpha$ mutants suggest that these responses are elicited by volatile compounds. Urine is notably a complex biofluid containing a large number of volatile compounds that differ in their chemical and physical features (i.e., molecular weight, polarity, hydrophobicity). The urine volatilome^33^, which includes the signatures of metabolic breakdown of food, contaminants, drugs, endogenous and bacterial by-products, is related to the state of health of the individual. Altered concentrations in the volatilome have been found in spectra of GC/MS on cancer biofluids^34–36^, but, to date, the identification of specific cancer biomarkers through these methods may be problematic given the sensitivity up to the micromolar range and the difficulties in identifying large percentages of the total metabolites which are detected in a biological matrix^37^. Among this high number of molecules, the high sensitivity and specificity of the *C. elegans* olfactory system (up to the nanomolar range^23^) may guide the identification of the essential metabolic signature of cancer.

In this work, we propose a worm-based strategy to discriminate between healthy and breast cancer urine samples by exploiting the high sense of smell of the nematode. We first examine the strong innate attraction of the nematode to urine samples from cancer patients. Specifically, calcium imaging analyses allowed us to measure a 97.22% accuracy of *C. elegans* ability in discriminating between urine samples collected from healthy subjects and women with breast cancer. We also prove that cancer samples activate specific olfactory neurons (AWCs) reliably, and identify a subset of GPCRs that may be involved in this neuronal response. A deep understanding of the biochemical mechanisms underlying the strong attraction towards cancer samples in *C. elegans* can generate valuable insights about the identification of essential biomarkers for early cancer detection.

## Results

### Sample collection and clinical characterization of breast cancer subjects

We collected n=36 urine samples from women with breast cancer and n=36 urine samples from sex- and age-matched healthy donors. Both groups ranged between 25 and about 90 years of age. Cancer features are reported in Table 1: 88.9% of cases are invasive ductal carcinoma, the most common form of breast cancer. To test the ability of *C. elegans* to detect cancer early, the majority of breast cancer patients (66.7%) were selected in the initial stages of the disease.

**Table 1:** Breast cancer (bc) type/staging in the analyzed cohort.

Tumor histology	Number (%)
Age [median (range)]	$68.1 \pm 11.9$
Gender (male/female)	0/36
**AJCC/UICC stage**	
0	2 (5.6%)
I	22 (61.1%)
IIIa	4 (11.1%)
IIIb	1 (2.8%)
IIIc	3 (8.3%)
IVa	1 (2.8%)
Undetermined	3 (8.3%)
**TNM classification**	
Primary tumor (T)	
Tx	2 (5.6%)
T0	2 (5.6%)
Tis	1 (2.8%)
T1	23 (63.9%)
T2	6 (16.7%)
T3	0 (0%)
T4	2 (5.6%)
Regional lymph nodes (N)	
Nx	1 (2.8%)
N0	26 (72.2%)
N1	5 (13.9%)
N2	4 (11.1%)
Distant metastasis (M)	
M0	34 (94.4%)
M1	1 (2.8%)
Mx	1 (2.8%)
**Histologic type**	
Invasive ductal carcinoma	32 (88.9%)
Invasive lobular carcinoma	4 (11.1%)

### Chemotaxis towards women urine samples is affected by the hormone cycle

**Figure 1:**
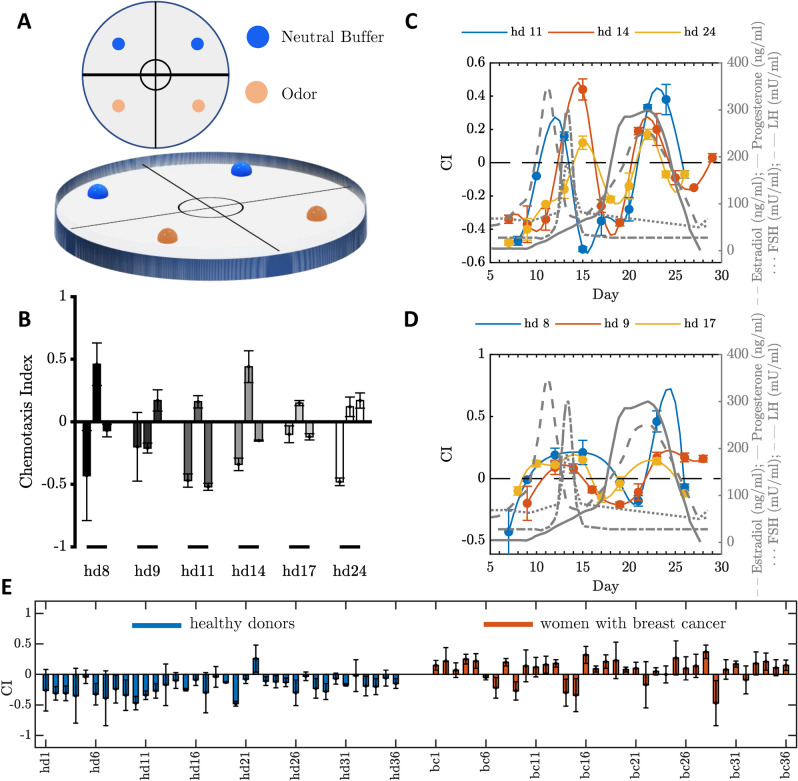
*C. elegans* chemotaxis assays with urine samples from healthy donors and cancer patients. (**A**) Population chemotaxis assay plate design. A 10 cm diameter Petri dish was used to conduct chemotaxis assays. The plate was divided into four quadrants, two odorant areas (+) and two control areas (−). One microliter of either cancer or control urine samples diluted at 10^−1^ was placed on the odorant areas (+). Nematodes were placed in the center of the plates and after 60 minutes a chemotaxis index (CI) was calculated as CI = (number of worms in the odorant areas (+) - number of worms in the control areas (−)) / total number of worms. (**B**) The chemotaxis index of *C. elegans* is influenced by female hormones. Chemotaxis assays on urine collected from six healthy donors (hd) during different days of the month showed variability in the chemotaxis index, suggesting an influence by female hormones. Error bars indicate SEM of three independent experiments performed on the same day on separate plates (**C,D**) *C. elegans* responses to urine collected from healthy donors are influenced by menstrual cycle rhythmicity. Chemotaxis assays were performed using urine collected from six healthy donors (hd), three times a week, starting two days after the end of the period, until the next period. CI correlates with LH, FSH and estradiol release preceding ovulation, and the progesterone peak during the luteal phase^38^. It is worth to notice that the pre-ovulation curve is narrower in group 1 (**C**) if compared to the curve of group 2 (**D**). Error bars indicate SEM of three independent experiments carried out on the same day on different plates. In (**C**,**D**), we included samples provided by three volunteers who were available to collect urine samples three times a week over one month. Grey curves indicate the fluctuations of hormone release during the menstrual cycle (reference graph modified from^38^). (**E**) Bar plot of the resulting chemotaxis index values for each sample of the control (blue bars) and positive group (red bars) reported with the corresponding standard deviation.

To confirm that *C. elegans* displays avoidance towards urine samples from healthy donors^32^, we initially performed population chemotaxis assays (Fig. 1A) using samples from healthy women. A first set of assays carried out on samples from 10 independently selected women showed contradictory results (Supplementary Fig. S1). Surprisingly, opposite chemotaxis indexes (CI) values were also observed in chemotaxis assays performed on independent samples collected from the same individual at different days along a period of one month (Fig. 1B). These data suggested a major role of the female hormone cycle in modulating the chemotactic response of nematodes. To test this hypothesis, we collected biological samples three times a week from six healthy fertile, pill-free women, three of them being <30 years of age (group 1), the others being >40 years (group 2). Sample collection started two days following the end of the menstrual cycle and finished at the beginning of the next. Data reported in Fig. 1C,D suggest a positive correlation between the chemotactic response of animals and both the follicular and periovulatory phases. While a peak in the estradiol release characterizes the former phase, the levels of the follicular stimulating (FSH) and luteinizing (LH) hormones promptly rise during the latter^38^. Similarly, a positive CI was measured during the mid-luteal phase, which is characterized by a second wave of estrogen release associated with the progesterone peak. Interestingly, the shape of the pre-ovulation peaks was narrower within group 1 (younger women) compared to group 2 (eldest women), in agreement with the well-established changes in hormonal release with aging^38^.

These findings demonstrate how the avoidance behavior of *C. elegans* towards control urine samples is strongly influenced by the menstrual cycle. Such a hormone-dependent behavior has not been previously reported^32^, making data interpretation quite puzzling. In subsequent analyses, samples of fertile women have been collected during two relatively narrow and specific time-windows, i.e., a few days after the end of the period or between the follicular and luteal phases, in order to avoid false positive results.

### *C. elegans* is attracted to urine samples from women with breast cancer but avoids control samples

Two cohorts were enrolled for this study: a first group included 36 women aged 38–92 years diagnosed with primary breast cancer (bc) as reported in Table 1; a second group was composed of 36 supposedly healthy and age-matched females (hd). Based on the aforementioned considerations, samples from fertile women were collected two days following the end of the menstrual phase (see Methods for details). In line with previous data indicating a dose-dependent effect of cancer-derived biological samples on *C. elegans* chemotaxis behavior^32^, we tested several concentrations of urine and found that the maximum attraction to breast cancer samples, as well as the highest avoidance towards controls, peaked at a dilution of 10$^{-1}$ (Supplementary Fig. S2). Population assays showed that *C. elegans* displays a significant preference for samples collected from women with breast cancer, whereas control urines behave as chemorepellents promoting avoidance (*P < 0.001; ANOVA, post-hoc Tukey’s HSD) (Fig. 1E and Supplementary Fig. S2). The sensitivity of the test was 75$\%$, while the specificity was 97.22$\%$, yielding an accuracy of 86.11$\%$. In two smokers, the analysis was also carried out after one smoke-free week, with no changes in the overall response, indicating that the result is not affected by cigarette smoking. These data confirm and extend previous findings indicating that *C. elegans* hermaphrodites can detect breast cancer urines and discriminate them from control samples with relatively high specificity and sensitivity.

### A major role of AWC^ON^ neuron in sensing cancer urine samples

**Figure 2:**
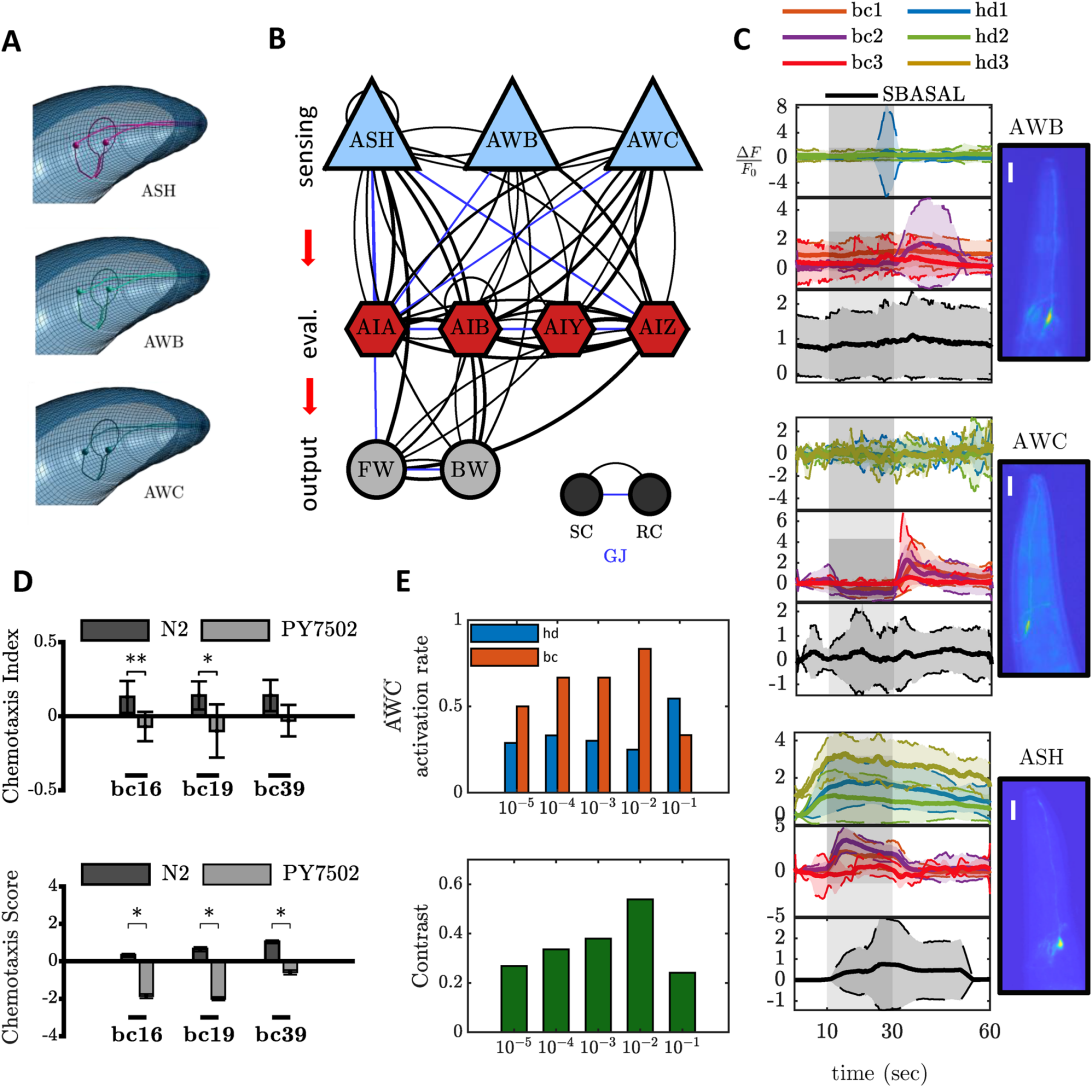
AWC is the main olfactory neuron mediating attraction towards cancer urine samples. (**A**) Head chemosensory neurons mediating aversive response (ASH and AWB) and attraction (AWC), with processes extending to the tip of the nose. (**B**) Olfactory sensory neurons, downstream interneurons, and motoneurons. (**C**) Mean calcium imaging traces normalized according to the standard deviation (N=6) from (top to bottom) ASH, AWB, and AWC neurons responding to control samples (repulsive), cancer urine samples (attractive), and to SBasal (neutral). Stimulation time is shaded in grey. AWC^ON^ neurons show the most reliable responses, making them the best candidate for comparisons with the CI. We show the results from the samples that we first collected and that were readily available. (**D**) AWC neurons sense cancer metabolites. Population (top) and single-animal (bottom) chemotaxis assays showed that AWC genetically ablated worms are unable to sense cancer metabolites. Bar plots indicate chemotaxis indices or chemotaxis scores in response to three informative cancer samples (bc16, bc19, and bc39). Error bars reported in population assays indicate SEM of three independent experiments carried out on the same day on different plates (*p <0.05; **p <0.002, Student’s t-test). Error bars of single-animal chemotaxis assays indicate SEM of several tested animals (bc16: N2= 25, PY7502= 28; bc19: N2= 28, PY7502= 29; bc39: N2= 31, PY7502= 27) (*p <0.05, Mann-Withney test). (**E**) The top graph reports the activation rate (y-axis) of AWC^ON^ neurons in response to removal of urine samples of cancer subjects (bc) or control samples (hd) for solutions with rising concentrations (from 10^−5^ to 10^−2^). The bottom graph shows the resulting contrast for each concentration. The concentration of 10^−2^ yields the highest activation rate (83.33%) and a reasonably good contrast between control and positive samples.

Chemotaxis is the downstream outcome of the integration of several upstream signals from chemosensory neurons. The result of a chemotaxis assay may be altered by the presence of interfering stimuli of any nature that may originate from temperature, humidity, and/or mechanical solicitations for instance. To effectively determine whether *C. elegans* perceives cancer metabolites and with what accuracy, calcium imaging proves to be a powerful tool because it allows to directly record the activity of upstream olfactory neurons activated by ligands regardless of the presence of concurrent cues sensed by other neurons. The dissection of the neural olfactory circuit in *C. elegans* through ablation, behavioral assays^23,24^, calcium imaging^39^, and the electron micrographs^16^ shows the existence of three main pairs of olfactory sensory neurons with winged cilia named AWA, AWB and AWC^40,41^. AWA and AWC mediate attraction to volatile odorants while AWB mainly responds to repulsive compounds^42^. The two AWC neurons are structurally similar but functionally different: AWC^ON^ neuron expresses a chemoreceptor-encoding gene, *str-2*, missing in AWC^OFF^, that instead expresses an alternative chemoreceptor gene, *srsx-3*. As a consequence, AWC^ON^ neuron senses 2-butanone and acetone, while AWC^OFF^ neuron senses 2–3 pentanedione. This genetic and functional asymmetry is fundamental in *C. elegans* odor discrimination and it could play a pivotal role in cancer sensing^43^. AWC olfactory neurons are activated by odor removal and inhibited in the persistent presence of attractants. Another neuron pair results to be a good candidate for mediating the avoidance behavior towards urine samples collected from healthy subjects: the ASH polymodal neuron pair, which is reported to be associated with aversive stimuli^23^. All these neurons are located in the head of the nematode (see the 3D reconstruction in Fig. 2A based on the data from^44^, available at the open source repository https://github.com/openworm/CElegansNeuroML). The olfactory neurons synapse onto several interneurons which act on motoneurons that initiate movements towards or away from the source of the stimulus (Fig. 2B).

A major role of AWC^ON^ and a minor one of AWA in mediating attraction towards diverse biological samples from different types of solid tumors has recently been shown^32^. To better understand which neurons participate in the chemotaxis behavior towards urine samples, we record the activity of AWA, AWB, AWC, and ASH in response to stimulation with cancer and control samples. To do this, we use transgenic worms expressing a calcium indicator on the neuron of interest, specifically GCaMP3 (circularly permuted green fluorescent protein-calmodulin-M13 peptide version 3). These animals are challenged against a selected panel of well-known attractive odorants to demonstrate functional equivalence with the wild-type N2 strain (data not shown). We then test the response of the aforementioned neurons to chemicals associated with either a strong negative chemotaxis index (for neurons sensing aversive stimuli) or a strong positive chemotaxis index (for neurons sensing attractants). As a reference for strong repulsion while assessing the response of the AWB and ASH neurons, we used a urine sample that tested negative multiple times in chemotaxis assays. As a reference for attraction for the AWC and the AWA neurons, we used three urine samples that tested positive multiple times in the assays. Figure 2C shows typical responses from three of the four candidate neurons. We exclude AWA neurons, being their role secondary, less reliable, and redundant when compared to AWC neurons, as also demonstrated in^32^. Moreover, the AWA neuron expresses a lower number of chemoreceptors^9^, if compared to those of AWC neurons with two receptors in common with AWC: this makes it sensitive to a lower array of chemicals^22^. Responses from the AWB neurons seem to not correlate with either addition or removal of attractant or repellent (although showing calcium-related dynamics during the experiment), while the ASH neuron responses were not reliable enough, showing a high standard deviation. The AWC^ON^ neuron instead responds with robust hyperpolarization during the addition of cancer urine and strong depolarization upon its removal in a stereotyped fashion while it is often silent with healthy control samples.

To further demonstrate the role of AWC^ON^ in recognizing breast cancer metabolites, we perform chemotaxis assays on the PY7502 strain, in which both the AWC neurons were genetically ablated. Since the chemotactic response is known to be affected by the population density on an assay plate^45^, odor preference of AWC-ablated animals is tested in both population and single-worm assays, by using three among the most informative cancer urine samples (i.e., those associated with the higher CI). As expected, and in line with previous findings, our data show that AWC-ablated nematodes have a reduced ability to sense cancer metabolites (*P < 0.05, Mann-Withney test), indicating an essential role for the AWC amphid neurons in mediating this behavior (Fig. 2D). Together, these results suggest that the AWC^ON^ neuron plays a determinant role in driving attraction towards cancer biofluids. However, because this neuron is associated with attraction, it cannot account for repulsion sensed by the nematodes.

### Specific concentration and stimulation time maximize the discriminating power of AWC^ON^

To measure the overall tendency of *C. elegans* to respond through the activation of the AWC^ON^ neuron upon the subtraction of a specific chemical, we define the neuronal activation index (NAI):

1 $$\begin{aligned} NAI=2 \left( \frac{N_{act}}{N_{tot}}-0.5\right) , \end{aligned}$$

where $N_{act}$ is the number of nematodes responding with the activation of the AWC^ON^ neuron upon subtraction of the odorant and $N_{tot}$ is the number of viable nematodes tested for the same chemical stimulus. This definition forces the index to range from $-1$ to 1 and allows us a direct comparison with the chemotaxis index (see Methods for details).

To define the optimal range of urine dilution at which the nematode sensitivity is maximized, we test various concentrations from 10^−1^ to 10^−5^ (the pure sample is discarded because urine pH may interfere with the nematode preferences) of a subset of samples eliciting either a highly positive or a highly negative index in chemotactic assays. The value that maximizes the activation rate upon positive sample removal is 10^−2^ (activation rate of 83.33%). At this value, the contrast between the activation rates of positive and control samples is also the best one for the considered range (Fig. 2E). To determine how *C. elegans* distinguishes cancer urine samples from control ones, we investigate the neuronal activation index of AWC as a function of the length of the stimulus time for a control sample. We record the AWC activation rates and measure the corresponding NAIs for control samples as a function of the length of the stimulus time (Supplementary Fig. S3). The NAI values highlight how the stimulus presented for longer times elicits attractive-like responses in the majority of the tested nematodes, whereas shorter time windows are associated with no response from most of the collected AWC traces. This suggests that metabolites eliciting the activation of AWC neurons are less concentrated in control samples in comparison with samples from cancer patients.

### Analysis of the activity of AWC neurons greatly improves the accuracy in cancer screening

**Figure 3:**
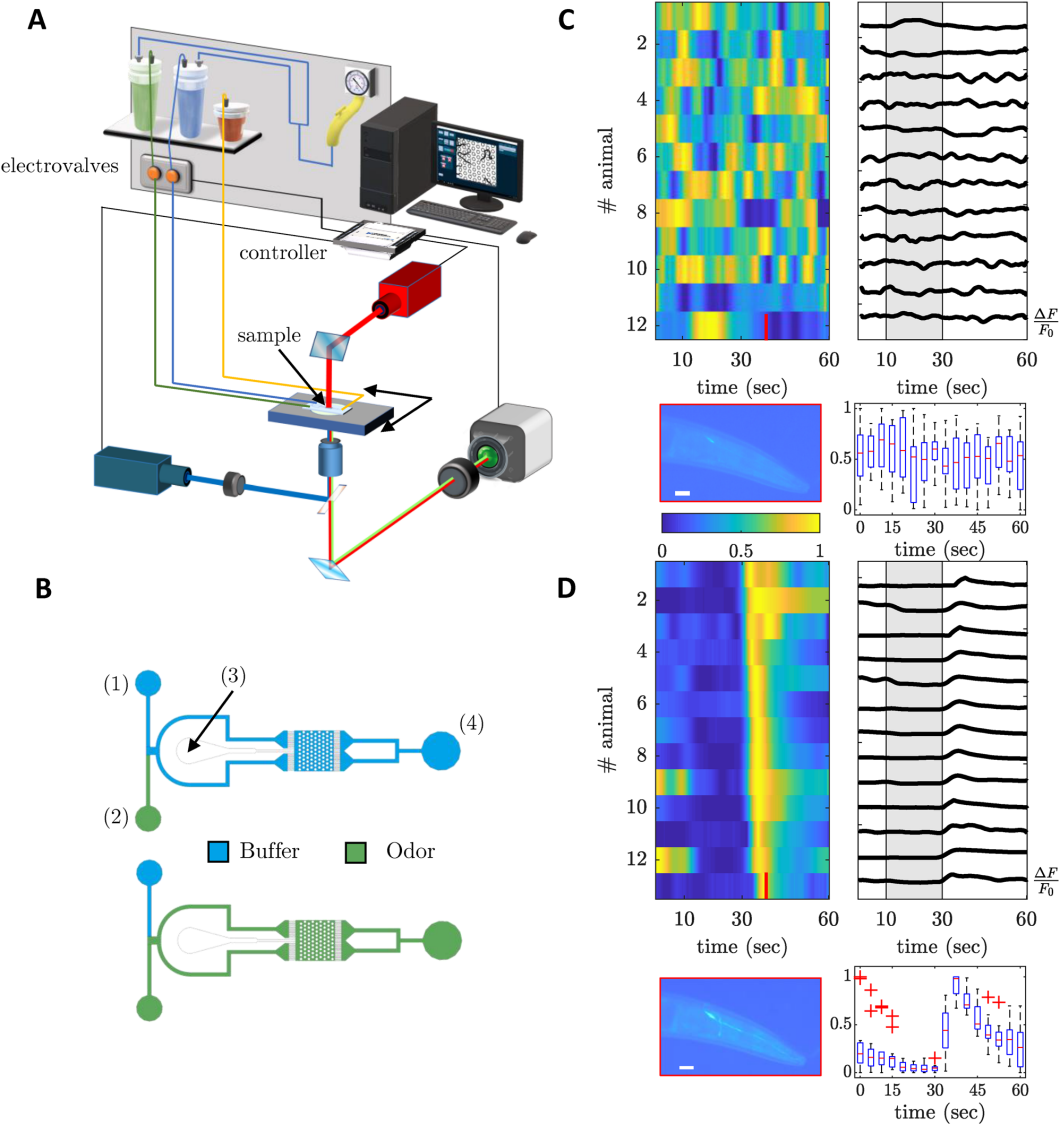
Wide-field imaging of neural activity upon chemical stimulation. (**A**) The sketch showing the experimental setup for simultaneous chemical stimulation via electro-valves and recording of neural responses via 470 nm blue excitation of GCaMP (blue line) and its emission (green line), and 660 nm light (red line) for transmission images. (**B**) Top view showing pulse arena geometry in two different conditions (buffer or odor filling). (**C,D**) Heatmaps and individual traces representing peak normalized neural responses ($\Delta F / F_0$) across 12 animals and time from (**C**) healthy donors and (**D**) cancer subjects recorded on AWC^ON^ neuron (pictures show a crop of a wide-field 4X fluorescence image on a head with a quiescent AWC^ON^ neuron (top) and a chemically stimulated one (bottom)). Box plots show the corresponding population average peak $\Delta F / F_0$. The central mark indicates the median, and the boxes indicate the 25th and 75th percentiles, respectively. The whiskers extend to the most extreme data points not considered outliers, and the outliers are plotted individually using the ‘+’ symbol.

For high-throughput experiments in calcium imaging, we simultaneously record neuronal activity from multiple nematodes. Nematodes are loaded onto a custom-designed microfluidic device with an all-liquid environment with a design based on the pulse arena of^46^ (Fig. 3A,B). We acquire results for 36 women with breast cancer and 36 healthy subjects. Each sample has been tested in at least two different sessions, in which a minimum of 25 worms has been exposed to the odorant. This means that the NAI reported for every subject is obtained considering a minimum of 50 nematodes. In Fig. 3C,D show typical traces obtained for one sample of both groups.

**Figure 4:**
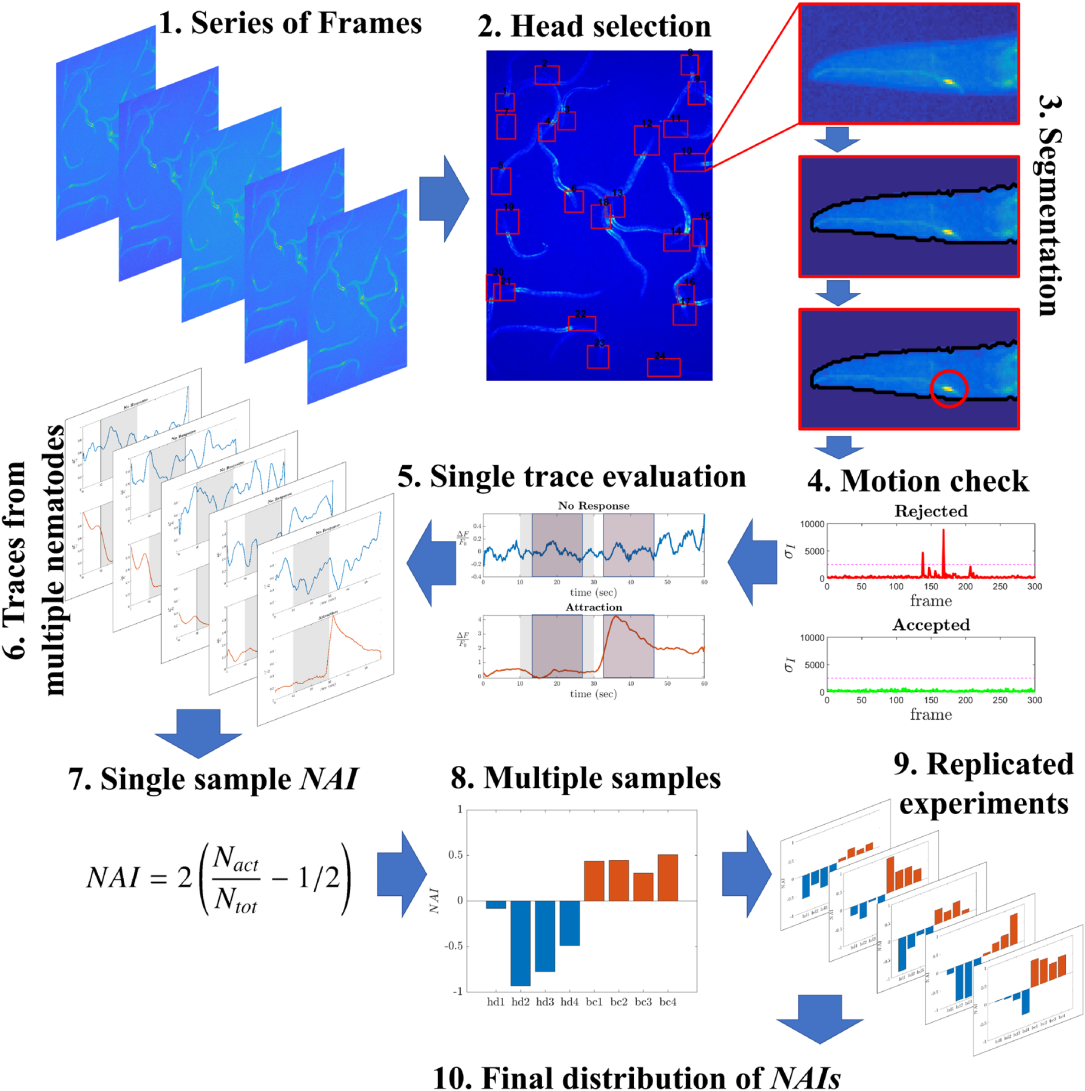
Pipeline of the post-processing processes. Starting from a series of frames (1), heads are selected and segmented for AWC^ON^ identification (2–3). If the nematode stays still during the acquisition, it will pass the motion check and the trace will be evaluated (4–6). The NAI is calculated considering all viable traces available in the acquisition (7). Each experimental run tests about 4 positive samples and 4 control ones (8). Averaging between different days of experiments (9) finally yields an average NAI and a standard deviation for each sample (10). More details are reported in the Methods section.

To calculate the NAIs, we define a rule for the systematic identification of significant activation events in the AWC^ON^ neuron. To do this, we employ a custom script that extracts the fluorescence traces from the videos and evaluates them. Figure 4 shows the pipeline of the post-processing process for an example image. More details are reported in the relative section of Methods.

**Figure 5:**
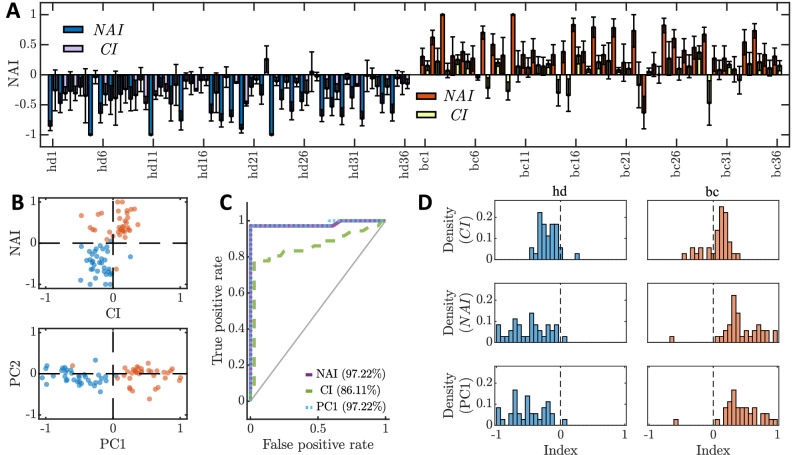
Comparison between the CI and the NAI. (**A**) Bar plot of the resulting NAI and CI values for each sample of the control (blue bars) and positive group (red bars) reported with the corresponding standard deviation calculated assuming a binomial distribution of the variables. Although both indexes show a clear tendency (positive values for cancer samples, negative values for the control group), the accuracy and the contrast associated with the NAI is considerably higher if compared to the CI. (**B**) The top graph reports the scatter plot in the original space of NAI vs CI for the averaged values obtained over replicate samples of each subject (patients in red and healthy donors in blue). The bottom graph reports the first two principal components (PC1, PC2) of the Principal Component Analysis (PCA) obtained for the data reported in the top graph. The outcome of the analysis shows that PC1 explains 90.95$\%$ of the total variance with NAIs contributing to most of the separation between the points of the two groups (representing a loading coefficient for PC1 of 97.31$\%$). (**C**) ROC curves obtained for the CI, for NAI, and the first component of the principal component analysis. It is clear from the graphs that the calcium imaging measurements yield a better discrimination between the positive group and the control one. The first component of the PCA improves the discrimination power of the NAI, although it does not differ from it too much. (**D**) Distributions of the measured CI, NAI, and PC1 (from top to bottom), for both the control group and patients (left and right graphs respectively for each row).

Figure 5A shows a bar plot reporting for each sample the resulting NAI (left) and the CI (right). As expected, there is a clear tendency in the NAIs obtained through calcium imaging: cancer samples are associated with a positive NAI, while healthy subjects have a negative NAI, meaning that the first group elicits the activation of the AWC^ON^ neuron as opposed to the second one, which does not trigger with the same frequency the chemical response in the neuron. Moreover, the measured NAIs are consistent with the results obtained in the chemotaxis assays, and with the assumption that the AWC^ON^ neuron mediates chemical attraction, contributing to a positive CI. PCA analysis (Fig. 5B, bottom graph) considering both indexes (Fig. 5B, top graph) shows a first component explaining the 90.97% of the total variance. The NAI contributes to more than the 97.31% of this component, highlighting its significance as a discriminator. The ROC curves as well, show how the NAI index is a better descriptor to discriminate between the two groups of samples (Fig. 5C) compared to the CI. When looking at the distributions of the CIs, the NAIs, and PC1 (Fig. 5D, from top to bottom respectively), the use of a linear combination of the two indexes (as PC1) contributes to the shifting of a few values close to zero in the NAI distribution. For the healthy donors distribution, this is probably because the NAI lacks a corresponding N_-_ term, accounting for repelled nematodes in the CI. This makes it more sensitive to detect attraction, while it is unable to distinguish between a lacking response and repulsion. Linearly combining the NAI with a quantity that takes into account repulsion as well, increases the distance from zero for some of the low-intensity values appearing in the NAI distribution. The accuracy associated with calcium imaging experiments (97.22%) is significantly higher than the one obtained through chemotaxis (86.11%). The accuracy is defined as the proportion of true positives and true negatives among the total number of cases examined. Additionally, the NAI index is associated with higher accuracy in discriminating urine samples of the cancer group from the healthy one. The fact that the accuracy of the NAI evaluated only on the AWC^ON^ neuron is higher than the one associated with the chemotaxis index supports the hypothesis that this neuron plays the main role in mediating attraction towards urine samples. For this to be true, urine samples eliciting attraction must contain chemicals at concentrations allowing them to interact with receptors on AWCs. This means that its receptors are good candidates as binding substrates for cancer-related metabolites, providing chemo-physical constraints on the interacting metabolites and their relative abundances. On the other hand, the high accuracy associated with the AWC^ON^ neuron also proves that chemical avoidance is not needed to discriminate between healthy samples and positive ones. However, *C. elegans* moves away from negative samples instead of just not showing attraction towards them. This suggests that there is a concurrent mechanism (yet not identified) contributing to the measured chemotaxis indexes, acting in a complementary way.

### Identification of the *C. elegans* G protein-coupled receptors involved in the attraction towards cancer samples

**Figure 6:**
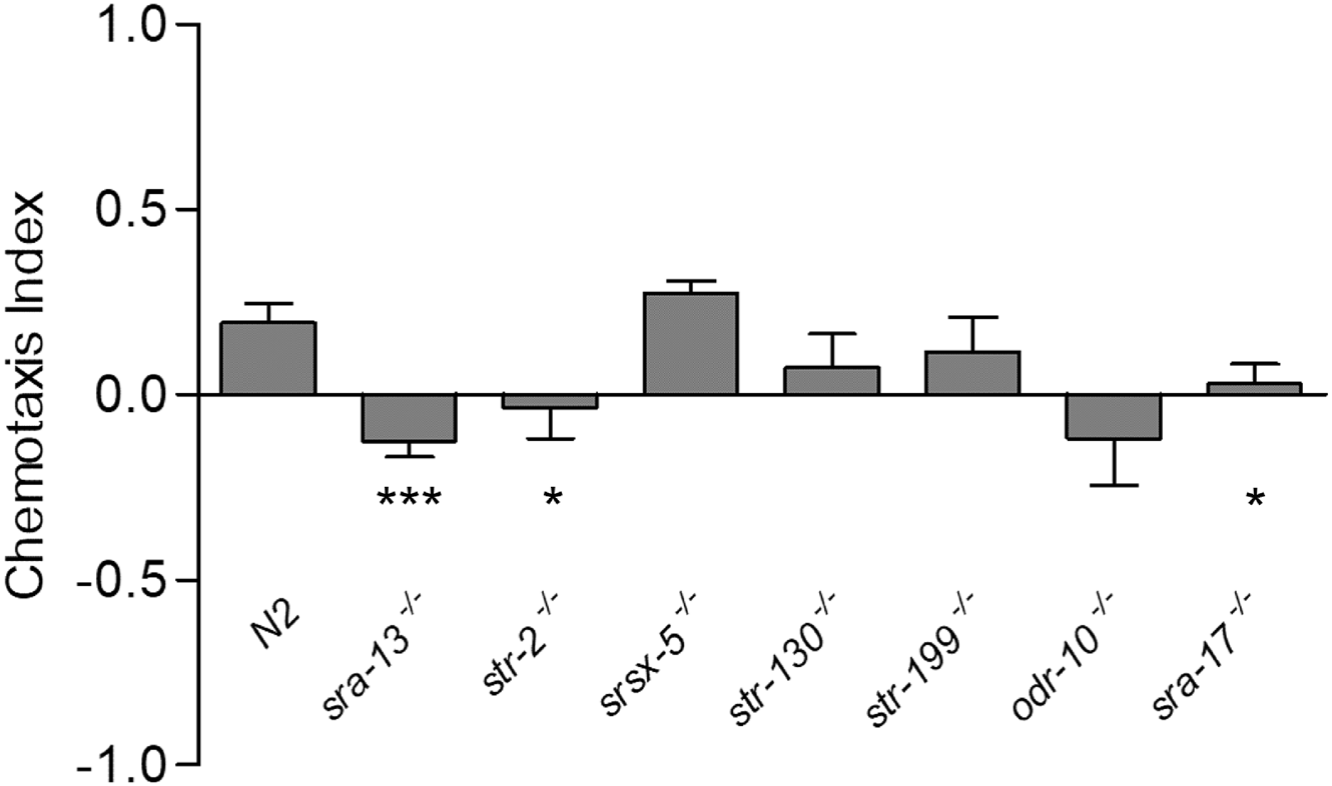
Chemotaxis index of animals harboring a deletion for the indicated GPCR gene. Strains whose CI was significantly lower if compared to that of N2 were considered as strong candidates for sensing breast cancer metabolites. Statistical significance was calculated using unpaired t-test with Welch’s correction. The graph is presented as the average of assays performed using two different urine samples (n$\ge$8 assays). Error bars represent SEM (*P < 0.05, ***P < 0.0001).

It is well established that AWA and AWC neuron pairs mediate positive chemotactic responses to volatile odorants^24^ and as the predominant response to cancer urine is positive chemotaxis, we assumed that at least one cancer-metabolite receptor is expressed in the AWA and/or the AWC neurons. To test this hypothesis and identify the putative G protein-coupled receptors (GPCRs) responsible for sensing cancer urine metabolites, a small-scale pilot screen of AWC/AWA-GPCR mutants available from the *Caenorhabidtis* Genetics Center (CGC), was performed via chemotaxis assays (Table 2). We chose to expose the mutant strains to two cancer urine samples. The ones that showed a significantly reduced CI compared to that of wild-type animals, were to be considered putative cancer-sensing GPCRs. We first focused on the mutant strains harboring a deletion of individual GPCRs expressed in AWC neurons. Five mutant strains were tested. Two of them, *sra-13(zh13)* and *str-2(ok3148)* resulted in a significantly lower CI compared to N2, suggesting an involvement of these receptors in responding to breast cancer urine samples (Fig. 6). *Str-2*, is expressed in AWC neurons exclusively, while *sra-13* is expressed in both AWA and AWC neurons (Table 2). Three null mutants, *srsx-5(gk960578)*, *str-130(gk948599)*, and *str-199(gk949542)*, displayed a CI towards breast cancer samples that was not significantly different compared to that observed in wild-type animals, indicating that the encoded receptors are unlikely to play a major role in sensing cancer metabolites (Fig. 6).

A couple of strains, namely *srt-26(gk947940)* and *sri-14(ok2865)*, could not be tested because, for the former, the deleted region encompasses a pseudogene, as described in WormBase release WS273, and the latter manifested locomotion defects in a thrashing assay (Supplementary Fig. S4) impeding proper completion of the chemotaxis assay, which requires normal motility to be performed. It is worth to notice that this finding may highlight an unpredicted expression of the *sri-14* gene in neurons controlling locomotion, although an off-target effect occurred during the original mutagenesis screen cannot be excluded. On a second instance, despite the lower performance of AWA neurons in responding to urine samples, the results observed with *sra-13(zh13)* mutant are suggestive of a putative contribution of such neurons and additional analysis on AWA-GPCR deletion mutants were therefore included. We examined a couple of strains available from the CGC, namely *sra-17* *(ve511[LoxP+myo-2::GFP+NeoR+LoxP])* and *odr-10(ky225)*. Interestingly, *sra-17* deletion mutant showed a significant diminished CI towards cancer urine compared to N2 (Fig. 6). This result suggests that despite the less robust calcium response of the AWA neurons, receptors on such neurons might have a role in recognizing breast cancer metabolites, confirming the complexity underlying the olfactory system of *C. elegans*. Major information on the genes encoding the receptors herein analyzed are summarized in Table 2.

**Table 2:** Summary of GPCRs functions and result in the chemotaxis assays. Information on expression profile and function of each receptor was acquired from WormBase version WS273. Comparison between N2 and mutant strains chemotaxis index is reported (p-value). *P < 0.05, ***P < 0.0001, ns = non significant.

Gene name	Neuron	Functions	p-value
*sra-13*	AWA-AWC	It exhibits olfactory receptor activity. Found to mediate negative regulation of RAS/MAPK cascade during olfaction of volatile attractants^47^	0.0001 (***)
*str-2*	AWC^ON^	It exhibits olfactory receptor activity. Required for attraction towards 2-heptanone^9^	0.03 (*)
*odr-10*	AWA	It exhibits olfactory receptor activity and binds the odorant diacetyl. As yet, this is the only GPCR in*C. elegans* whose specific ligand has been determined^48^	ns
*sra-17*	AWA	It is predicted to have G protein-coupled receptor activity	0.04 (*)
*str-130*	AWC^OFF^	It is predicted to encode a protein with the following domain: 7TM GPCR, serpentine receptor class r (Str)	ns
*str-199*	AWC	It is predicted to encode a protein with the following domain: 7TM GPCR, serpentine receptor class r (Str)	ns
*srsx-5*	AWC	It is predicted to have G-protein coupled receptor activity	ns
*sri-14*	AWC	It is required for detection of chemical stimulus. It senses the odorant diacetyl at high concentration in ASH neurons fostering avoidance responses^25^	Not tested (motility defects)
*srt-26*	AWC	Pseudogene. Expression was detected in the intestine and the nervous system	Not tested (pseudogene)

## Discussion

In this study, we reported the ability of *C. elegans* to efficiently discriminate between subjects diagnosed with breast cancer and healthy controls by responding to urine, a bio-fluid that harbors an odor signature that is cancer-specific^32^. The strongest evidence showing that *C. elegans* detects cancer smells in urine resides in the nematode binary behavior observed in chemotaxis assays and calcium imaging analyses. Indeed, we measured that urine from cancer subjects induced attraction while avoidance was observed towards control samples with high accuracy (>97%). We also showed that the response of the nematode is dependent on the female hormone cycle, a phenomenon that has not been previously reported^32^. According to these findings, we suggest collecting urine samples from fertile women during two specific time-windows, i.e., a few days after the end of the period or between the follicular and luteal phases, to lower the rate of false positives in the context of a diagnostic setting.

Genetic analyses allowed us to identify the sensory neurons involved in this behavior as well as the individual GPCRs potentially responsible for the recognition of urine cancer metabolites. Chemotaxis assays with mutant strains lacking AWC neurons (AWC-killed strains), implied the involvement of AWC olfactory neurons in attraction towards cancer urine samples. Even though the mutation of *ceh-36* affects a second chemosensory neuron, ASE, we could not confirm its contribution in sensing cancer urine as ASE is involved in the tasting response towards water-soluble metabolites. To measure a response towards this type of molecules, a different chemotaxis assay protocol is required^24^. We performed the experiments over the period of one hour, which is not enough time for water-soluble molecules to diffuse on the plate and be detected by the worms. A one-hour long chemotaxis assay is sufficient for volatile components to diffuse and to be sensed. Under these circumstances, we could not define a role for ASE neurons in sensing cancer urine. Nevertheless, the *ceh-36* mutants retained some capacity to discriminate between cancer urine and control samples suggesting that AWC is not the sole neuron detecting cancer smells. A possible contribution may derive from another olfactory neuron, AWA, also implicated in attraction towards volatile molecules^23^. However, calcium imaging analyses showed a less robust and accurate activity of this neuron if compared to AWC in response to urine samples.

To further dissect the molecular basis of this response, we investigated the role of GPCRs which are responsible for odor sensing. *C. elegans* sensory neurons express several GPCRs as opposite to mammalian neurons, in which one neuron only expresses one type of receptors. AWC neurons express more than twenty different kinds of GPCRs with unknown functions^22^. Chemotaxis assays using AWC specific GPCR knock-out mutants showed involvement of two of these GPCRs, namely, *str-2* and *sra-13*, suggesting a role for these receptors in recognizing cancer-related molecules. AWA neuron expresses approximately half the amount of the olfactory receptors expressed in AWC^22^. Worms harboring a deletion for *sra-13* and *sra-17*, both expressed in AWA, resulted to be defective in attraction towards cancer urine implicating that AWA olfactory neurons are also involved in cancer smells attraction. Of note, the molecular mechanisms underlying chemotaxis towards cancer metabolites are predicted to be more complex due to the possible synergistic effect exerted by multiple molecules. Furthermore, the analysis was restricted to GPCRs for which knock-out mutants were available from the *Caenorhabditis* Genetics Center. To explore the contribution of additional receptors, future studies will be directed to generate novel null mutants of genes encoding GPCRs expressed in AWC and/or AWA sensory neurons via CRISPR-Cas9 genome editing. Control urine avoidance behavior should also require G protein signaling pathways. However, ASH and AWB neurons, the main neurons mediating repulsion to volatile molecules, do not seem to induce avoidance from control urine. In fact, their neuronal dynamics are not reliable when the animals are exposed to control samples. The avoidance response from control samples could be the result of a complicated neuronal interaction involving several sensory neurons and a sophisticated signal transduction apparatus associated with possible co-expression and heterodimerization of GPCRs^49^ and could be more context-dependent if compared to the cancer attractive behavior. The biochemical complexity in the chemoreceptors expression may be an evolutionary adaptation mechanism to counterbalance the neuro-anatomical simplicity of *C. elegans* nervous system and its low plasticity^50^. As a result, this complexity makes it more difficult to address the role of single olfactory neurons and to fully identify receptor complexes and their mechanisms in the overall functioning of the corresponding neuron^51^. Nevertheless, the impressive accuracy (97.22%) of AWC neurons suggests that the cancer urine blend comprises several components/metabolites present in distinctive relative abundances that robustly bind the olfactory receptors on these neurons.

In conclusion, our results confirm that *C. elegans* can sense cancer-related metabolites in urine samples collected from women with breast cancer. This ability is strongly dependent on the hormonal cycle, and we identify the time window of screening validity. We demonstrate that calcium imaging on olfactory neurons yields more reliable results in comparison with chemotaxis assays (86.11%), aside from being less time-consuming. Additionally, AWC responses in calcium imaging do not seem to be correlated with tumor stage, i.e., detection accuracy is high even at early stages (data not shown), whereas chemotaxis assays show a positive trend with respect to cancer stage (Supplementary Fig. S5), although the data distributions cannot be associated to a significant correlation. We verified through calcium imaging that the main contributor to the *C. elegans* cancer discriminating behavior is the AWC^ON^ chemosensory neuron and found a set of relevant GPCR receptors via genetic screens. The involvement of multiple GPCRs in the process suggests that multiple metabolites are sensed by *C. elegans* through the AWC neuron. However, it has to be highlighted that diverse GPCRs can also bind the same metabolite with different affinities.

Taken together, our results represent a proof of principle study for the exploitation of the incredible accuracy of the *C. elegans* chemosensory circuit to investigate the metabolic trace of cancer present in urine samples. To date, we do not have enough evidence to claim that a worm-based cancer detection strategy can provide a direct identification of the type of tumor. As for now, such a tool would be able to detect whether a cancerous condition exists but further analysis would be necessary to determine which type of cancer is spreading. We hypothesize that different cancer types potentially produce diverse odors perceivable by the nematode through its olfactory receptors (GPCRs). Our speculation is that different GPCRs would bind to different cancer-specific odors. Additional experiments exposing mutant worms lacking individual GPCRs to samples deriving from different cancer types would help us elucidate whether the worms can specifically discriminate against the cancer types. Moreover, the combined application of GC/MS analysis would provide a compelling “cancer odor profile” for different cancer types. In conclusion, the small nematode *C. elegans* holds the potential to help design a fast and cost-effective diagnostic screening test for breast cancer, with high reliability, based on the simple collection of biofluids.

## Methods

### Study design and sample collection

This study was approved by the Santa Lucia Foundation Ethics Board. All methods were carried out in accordance with relevant guidelines and regulations. Written and informed consent was obtained from all participants before study enrollment. Midstream urine samples were collected from subjects with diagnosed primary breast cancer (n=36) before surgical procedure at the M. G. Vannini hospital in Rome. These women were not hospitalized until the day of surgery, therefore they were not subjected to any specific dietary plan. All cases were correlated with histology findings. Controls (n=36) were healthy, age-matched volunteers with no declared history of pathological conditions. For both groups, pregnant women were excluded from study enrollment, as were women taking the contraceptive pill. Women who consumed alcohol a day before collection and subjects under bacteria, viral, or fungal infection were also excluded. All volunteers were not following a specific pharmaceutical treatment and were not required to follow any particular diet. For fertile women, samples were collected two days after the end of the menstrual cycle. Upon arrival, all urine samples were centrifuged for 15 min at 4 $^\circ$C, aliquoted in 0.2 ml tubes as single-use samples and stored at $-80\,^\circ$C until measurements and analysis. On the day of the experiments, urine samples were diluted in either S-Basal for calcium imaging or water for chemotaxis assays. A 1:100 dilution was used for calcium imaging measurements while 1:10 dilutions were utilized for odor screening chemotaxis assays. All dilutions were filtered before presenting them to the animals.

### Worm culture and strains

*Caenorhabditis elegans* strains were cultured at 20 $^\circ$C on nematode growth medium (NGM) plates seeded with *Escherichia coli* OP50 as food source (Brenner, 1974). Wild-type animals used for this study were the Bristol N2 strain, obtained from the *Caenorhabditis* Genetics Center University of Minnesota, Minneapolis, MN, USA, along with a number of deletion mutant strains, including VC2123: *sri-14(ok2865)* I, CX3410: *odr-10(ky225)* X, AH159: *sra-13* *(zh13)* II, RG3011: *sra-17(ve511[LoxP+myo-2::GFP+NeoR+LoxP])* II, VC20435: *srt-26(gk947940)*, VC30151: *srsx-5(gk960578)*, VC10129: *str-199(gk949542)*, VC40389: *str-130(gk948599)*, RB2316: *str-2(ok3148)* V. The strain harboring GCaMP in AWC^ON^ neuron was the PS6374: AWC^ON^-GCaMP syEx1240 *[str-2::GCaMP3+pha-1]; pha-1(e2123ts); him-5 (e1490)*; in ASH neuron was the PS6386: ASH-GCaMP syEx1246 *[sra-6::GCaMP3+pha-1]; pha-1(e2123ts)*; in AWB neuron was the PS6384: AWB-GCaMP syEx1245 *[str-1::GCaMP3+pha-1]; pha-1(e2123ts)*. The three of them were a kind gift from Dr. Alon Zaslaver, (The Hebrew University of Jerusalem, Jerusalem, Israel). The AWC-killed strain: PY7502, oyIs85 *[ceh-36p::TU813 + ceh-36p::TU814 + srtx-1p::GFP + unc-122p::DsRed]* was a kind gift from Dr. Arantza Barrios (The University College London, London).

### Behavioral assays

For the chemotaxis assays, ten adult hermaphrodites were picked onto fresh NGM plates seeded with OP50 *E. coli* bacteria. The worms were left to lay eggs for five hours and then removed from the plates. Eggs were incubated at 20 $^\circ$C and after four days adults were used for the test^52^. Chemotaxis assays were performed as previously described (Bargmann et al., 1993) with minor modifications. Briefly, assay plates were prepared using 10 cm Petri dishes containing 11 ml of modified NGM (2$\%$ agar, 5mM KPO4 [pH6], 1mM CaCl2, 1mM MgSO4). Adult worms were washed twice in S-basal, once in distilled water and approximately 50-100 worms were placed in the center of the plate right before starting the assay. The plates were divided into four quadrants referred to as the control and the odorant areas (Fig. 1A). The control areas contained 1 $\mu$l of neutral buffer, whereas the odorant area contained 1 $\mu$l of either control or cancer urine sample, diluted in Milli-Q water at 1:10. One microliter of 1 M $\hbox {NaN}_3$ was applied on each spot in order to immobilize the worms once they reached the area. After 60 minutes, animals were counted and a chemotaxis index (CI) was calculated as follows: (number of animals in the odorant area) – (number of animals in the control area) / total number of animals tested. Chemotaxis assays were performed in triplicate on separate chemotaxis plates, to ensure assay reproducibility^53^.

### Single worm chemotaxis assay

Single-animal chemotaxis assays were performed as previously described^54^ with some modifications. Assay plates were prepared using 5 cm Petri dishes containing 4 ml of modified NGM (2% agar, 5mM KPO4 [pH6], 1mM CaCl2, 1mM MgSO4). To visualize the trace of worms, the agar surface was dried for two hours just before placing the animals by opening the plate lid in the fume hood. Animals were placed on NGM plates without bacteria for one hour before the assay, then placed in the center of the assay plate, 2 cm away from the source of urine samples. One agar plug was placed onto the lid of the plates and 1 $\upmu$l of diluted sample was added to the plug immediately following the worm. After 20 min, the tracks left by the worms were visualized and the chemotaxis score was calculated as the sum of scores of the sectors through which the animal had traveled^54^.

### Thrashing assay

The locomotion behavior was assessed through thrashing assays performed on wild type worms (N2, Bristol) and *sri-14 (ok2865)* mutant animals. Assays were carried out at 20 $^\circ$C on 35 mm OP50-free plates filled with 1 ml of M9 buffer (6 g/l Na 2 HPO 4 , 3 g/l KH 2 PO 4 , 5 g/l NaCl, 0.12 g/l MgSO 4). From plates containing synchronized young-adults, one animal at a time was transferred to a food-free NGM agar plate for 2 minutes to remove the bacteria and then assayed after 1 minute of acclimatization. Thrashes were counted for 20 seconds and multiplied by 3 to obtain an estimate per minute. A single thrash was defined as a complete change in direction of bending at mid-body. At least 10 animals per genotype were assayed.

### Microfluidic device design for neuronal imaging

For calcium imaging experiments, odor pulses were delivered through microfluidic devices whose geometry is based on the pulse arena of^55^, with some variations introduced in the design: smoothing of the loading channel to facilitate nematode loading, removal of the free space between the layer of pillars and the inflow/outflow channels to avoid nematode piling up in the outflow side of the chip even under very slow flows, miniaturization of the overall dimension of the arena from 2 $\times$ 2 $\hbox {cm}^2$ to 3.25 $\times$ 3.25 $\hbox {mm}^2$ to record signals from approximately 20 animals at once within a field of view of 3.25 $\times$ 3.25 $\hbox {mm}^2$ at 4$\times$ magnification of the CMOS camera. The chip, placed on an XY-microscope stage, is linked to two pressurized bottles through Tygon tubes that drive the liquid directly in the inlet of the chip. The bottles are supplied by a gas line controlled with a gas adjustable regulator fixed at 100 mbar of pressure. The pressure value is validated to ensure a correct compromise between a fast odor switching in the arena (less than two seconds) and the avoidance of undesirable strong mechanical perturbations on the nematodes. Flow switch between odor and buffer is actuated slowly (about 2 seconds) by motorized valves directly controlled through software to prevent the inception of dangerous shock waves propagating in the tubes typically generated by a fast clogging of conventional solenoid valves. The electric command sent to the motorized valve is a pulse-width modulation (PWM) signal that allows to close them slowly without excessive stresses on the tube.

### Microfluidic device fabrication

The microfluidic device is prepared by using the soft lithography process^56^. The SU-8 (MicroChem 3000 series) structure is fabricated on a glass substrate by conventional photolithography to obtain a 50 microns thick monolayer microfluidic network. The high-resolution photomask has been directly printed on the microfluidic network with a laser writer to ensure a resolution of 3 microns at the minimum feature size. Then, the 1:10 PDMS (Polydimethylsiloxane Silgard 184) mold replica is cured after the casting process and the holes for inlet/outlet are made with a 0.6 mm puncher to subsequently connect with external Tygon tubes. The PDMS layer is bonded on a microscope glass slide by using an air-plasma treatment (HARRIK PLASMA) and thermal recovery with a hot plate. We first design the microfluidic network with CAD software and then produce the laser drawing of a high-resolution glass photomask.

### Experimental setup for calcium imaging

Calcium imaging recordings were made using a custom-designed inverted microscope. A low magnification objective with a high-NA (4$\times$/0.28 N.A.) and a sensitive low-noise CMOS camera with a large sensor area (13 $\times$ 13 $\hbox {mm}^2$) allow to collect fluorescence signals from approximately 15–20 nematodes at once in a field of view of approximately 3.25 $\times$ 3.25 $\hbox {mm}^2$. Excitation light was reflected on the sample from a high power LED (470 nm - M470L2, Thorlabs, Newton, New Jersey) with a FITC excitation filter (MF475-35, CWL = 475 nm, BW = 35 nm, Thorlabs) and a condenser (ACL2520U-A, Thorlabs), using a dichroic mirror (MD498, Thorlabs). The signal was collected by a digital CMOS camera (ORCA-Flash4.0 C11440, Hamamatsu, Hamamatsu City, Japan) through the dichroic mirror with a pass-band FITC/TRIC filter (59004x, Chroma, Bellows Falls, Vermont) using a 20X objective with a numerical aperture of 0.28 (XLFLUOR4X/340, Olympus, Tokyo, Japan). Before starting the recordings, a transmission channel (illumination at 660 nm through a high power LED, M660L4, Thorlabs) allowed to select the visualized area of the arena through a motorized stage (MLS203-1, Thorlabs). To reduce phototoxicity and prevent photo-bleaching, the excitation LED and the camera shutter were synchronized so that the arena was illuminated only during the exposure time (100 ms). During the acquisition, buffer flows for the first 10 and last 30 seconds, while urine sample flows between these time windows for 20 seconds. The LEDs, the electrovalves, and the camera were connected to a PC (Windows 10, 64-bit, Microsoft, Redmond, Washington) through a National Instruments controller (PCI-6221, National Instruments, Austin, Texas). A custom-made software in MATLAB (Mathworks, Natick, Massachusetts) was used for synchronized illumination, image acquisition, fluid delivery control, and data recording.

### Ca^2+^ imaging data analysis

All steps of calcium imaging data analysis are made through custom MATLAB scripts. Acquired images are pretreated by subtracting a mean background averaged over 20 background frames evenly recorded before and after the acquisition, and by applying an averaging filter for noise reduction. A custom made GUI allows the user to select ROIs containing viable nematode heads in the frame acquired 2 seconds after removal of chemical stimulus, in which the probability of having visibly active neurons is higher. Nematodes that appear to have restrained access to the surrounding chemicals are discarded. In each ROI, the user is required to select the position of the AWC neuron. On the basis of this information, the script tracks neuronal positions throughout the video in both temporal directions by looking for the maximum intensity averaged over a $5\times 5$ pixels area that may not be more than 30 pixels away from the neuronal position assigned in the previously processed frame. The resulting signal intensity at frame *i*, $I_i$, is calculated as $I_i=\frac{\Delta F_i}{F_0}$, where $\Delta F_i=F_i-F_0$. $F_i$ is the mean intensity of the segmented object representing the neuron. $F_0$ is the baseline value of fluorescence evaluated as the average of $F_i$ for $i=1,2,..10$, a stimulus free time window, in which the neuron is quiescent. In case a neuron changes position in the ROI from a frame *i* to its next one ($i+1$), the element-wise sum of the intensity difference, $ID_{i,i+1}$, between the ROIs in the two frames will vary much more if compared to a still video. Therefore, viable traces are identified as those ones in which the aforementioned difference satisfies the following condition: $ID_{i,i+1}<\overline{ID_i}+\sigma _{ID_i}$, where $\overline{ID_i}$ and $\sigma _{ID_i}$ are the mean value and the standard deviation respectively, calculated over all the *ID*s of the frames available in the range going from $i-20$ to $i+20$. This allows to exclude signals of moving neurons, whose evaluation may be affected by artifacts. All viable signals detected are visually validated by the user.

### Ca^2+^ data quantification

To quantify the activation rate of the AWC^ON^ neurons upon removal of chemical stimulus from calcium imaging traces, we defined the neuronal activation index (NAI) as, $NAI=2 (\frac{N_{act}}{N_{tot}}-0.5)$, where $N_{act}$ is the number of nematodes responding with the activation of the AWC^ON^ neuron upon subtraction of the odorant and $N_{tot}$ is the number of viable nematodes tested for the same chemical stimulus. The subtraction of 1/2 forces symmetry around zero, while the prefactor 2 grants that $\left| {NAI}\right| \le 1$, projecting the quantity into the range $\left[ -1, 1\right]$. From its definition, it follows that when $NAI>0$, the majority of the tested nematodes experienced AWC^ON^ activation, while a negative value is associated with the activation in a minority of them. For the systematic identification of significant activation events in the AWC^ON^ neuron, a custom MATLAB script evaluates the calcium imaging signal intensity, *I*, for each neuron. If the difference between the mean signal intensity in a 10 second-long post-stimulus time-window, $I_{off}$, and the mean signal intensity in a 10 second-long time-window while on stimulus, $I_{on}$, is three times higher than the standard deviation of the signal in the time-window while on stimulus, $\sigma _{on}$, the response is associated with activation. It is associated with a lack of response otherwise. All associations are then visually validated.

## Supplementary Information

Supplementary Information.
