# Supplementary material for: C. elegans-based chemosensation strategy for the early detection of cancer metabolites in urine samples
Source: Sci Rep. 2021 Aug 24;11:17133. doi: 10.1038/s41598-021-96613-z (PMC8385061; doi:10.1038/s41598-021-96613-z)
Supplement: Supplementary file 1 — Supplementary Information. [file 41598_2021_96613_MOESM1_ESM.pdf]

Supplementary Information

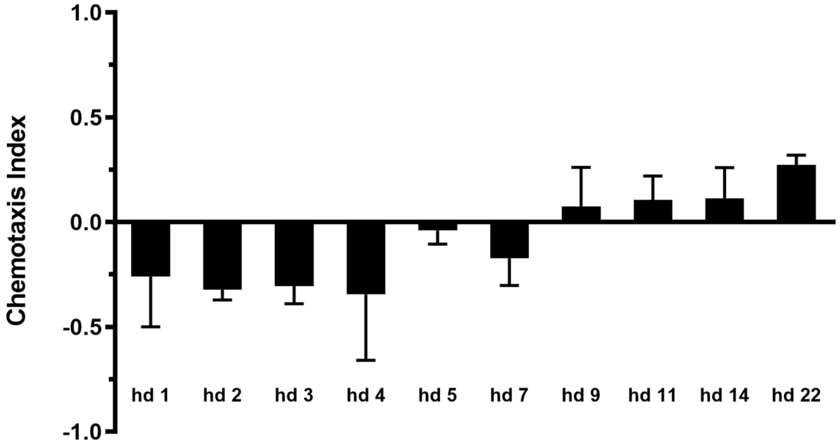

Supplementary Figure S1: **A first set of assays showed contradictory results.** Population chemotaxis experiments were performed on samples collected from different healthy individuals (hd). Results revealed a variability in the *C. elegans* chemotactic response, suggesting an influence by female hormones. Error bars indicate SEM of at least three independent experiments.

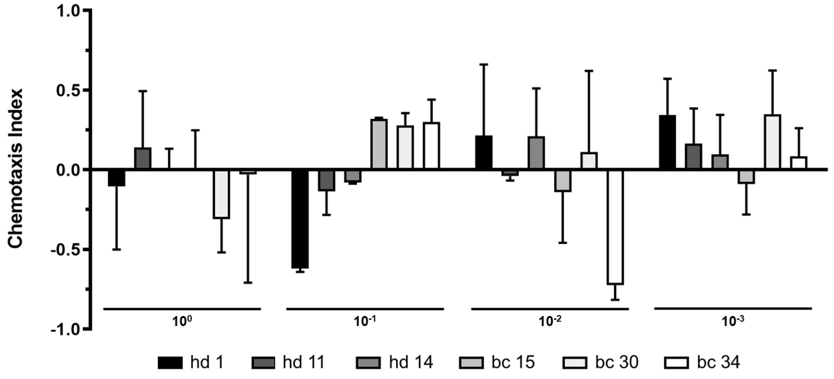

Supplementary Figure S2: **The 1:10 dilution was found to be the best concentration to discriminate between bc urine samples and controls.** Several concentrations of urines were tested via population chemotaxis assays. The maximum attraction to breast cancer samples (bc), as well as the greater avoidance towards controls (hd), peaked at a 1:10 dilution. Error bars indicate SEM of three independent experiments.

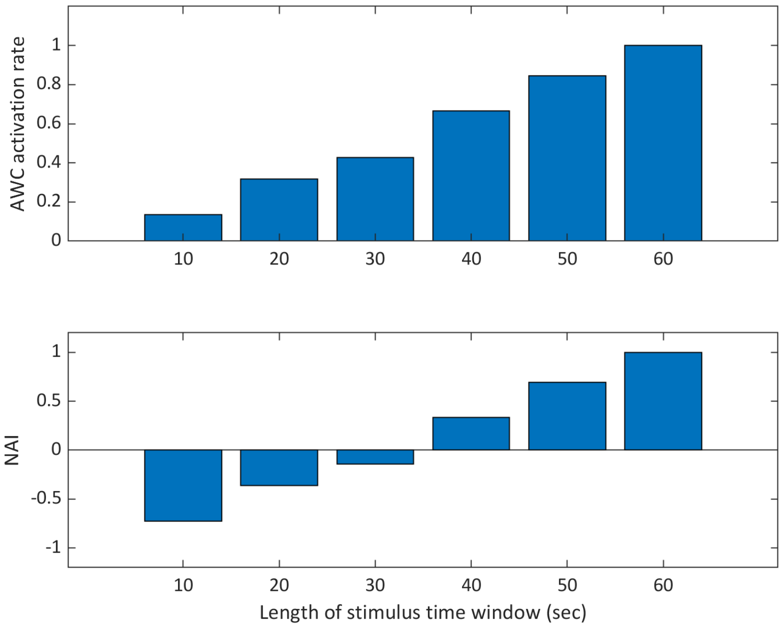

Supplementary Figure S3: **Preference inversion for control samples in AWC depends on the exposure time.** AWC activation rates and corresponding NAIs obtained for the same control sample as a function of the length of the stimulus time (top graph and bottom graph respectively). The NAI highlights how the stimulus presented for 40 seconds or longer times elicits attractive-like responses in the majority of the tested nematodes, whereas shorter time-windows are associated with no response from most of the collected AWC traces.

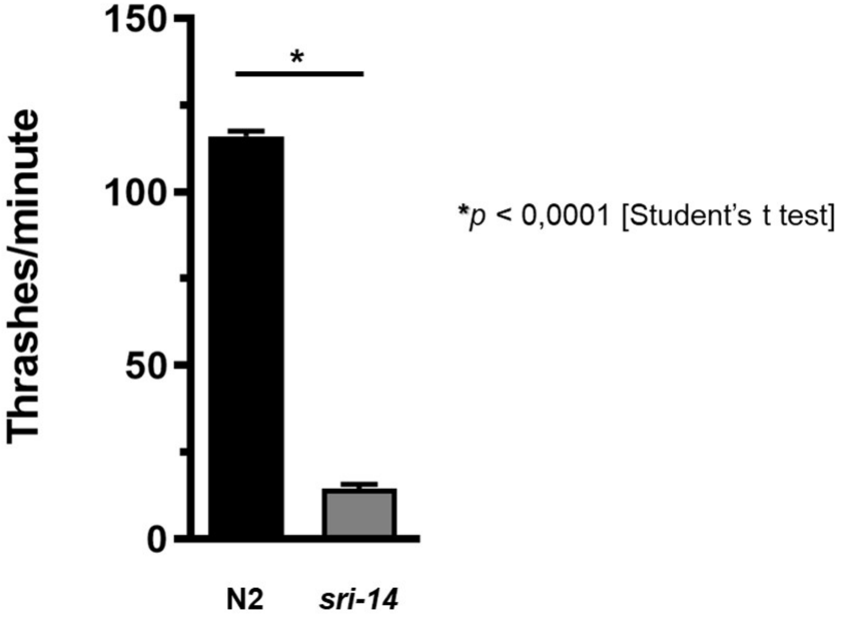

Supplementary Figure S4: **Mutant animals carrying a deletion in the sri-14 gene exhibited an uncoordinated phenotype**. Thrashing assays confirmed the locomotion defect observed in animals knock-out for the sri-14 gene. Mutant animals had a significant reduction in thrashes per minute compared to wild-type worms (N2) (*p $<$ 0.0001, Student's t-test). Error bars represent SEM of 15 different animals tested.

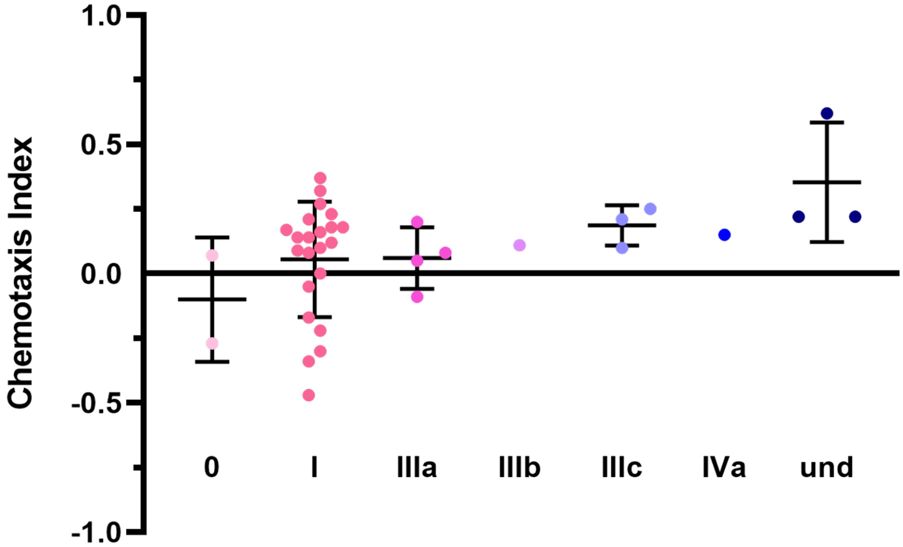

Supplementary Figure S5: **Chemotactic response as a function of tumour stage.** The nematode preference for bc urine is higher for more advanced stages of cancer, displaying a positive trend of the chemotactic index if compared to the cancer stage. The labels 0, I, IIIa, IIIb, IIIc, and IVa refer to the stage of the tumor, while und indicates undetermined stages. Each sample was tested in at least three independent experiments. Error bars indicate SD.
